# Differences in Lifestyle-Related Behaviors Among Healthy Weight, Overweight, and Obese Groups: A Secondary Analysis of Data on 4714 Adults in Poland

**DOI:** 10.3390/nu17132083

**Published:** 2025-06-23

**Authors:** Radosław Sierpiński, Mateusz Jankowski, Filip Raciborski

**Affiliations:** 1 Faculty of Medicine, Collegium Medicum, Cardinal Stefan Wyszynski University, 01-938 Warsaw, Poland; 2 Department of Population Health, School of Public Health, Centre of Postgraduate Medical Education, 01-826 Warsaw, Poland; 3 Department of Prevention of Environmental Hazards, Allergology and Immunology, Faculty of Health Sciences, Medical University of Warsaw, 02-007 Warsaw, Poland

**Keywords:** diet, dietary habits, nutrition, lifestyle, weight control, epidemiology

## Abstract

**Background/Objectives**: Excess body weight is a global public health problem. This study aimed to identify differences in food-related behaviors and physical activity among healthy weight, overweight, and obese adult individuals in Poland. Particular attention was paid to health inequalities among analyzed groups. **Methods**: This was a secondary data analysis of a dataset generated by the public institution in Poland, within a representative cross-sectional study among working adults (aged 18–64 years) in December 2024. A total of seven different questions on lifestyle-related behaviors were analyzed. **Results**: A total of 4714 adults were included in the analysis, of which 47.0% had a healthy weight (BMI 18.5–24.9), 34.2% were overweight (BMI 25–29.9), and 18.9% were obese (BMI 30–39.9). People aged 45–64 had almost 6 times higher odds of belonging to the overweight group compared to people aged 18–24 (OR = 5.7; 95% CI: 4.34–7.49). Men were more likely to be overweight (OR = 1.69; 95% CI: 1.47–1.95), as were rural residents (OR = 1.21; 95% CI: 1.05–1.39). The overweight group was more likely to monitor the number of steps taken during the day (OR = 1.42; 95% CI: 1.19–1.71) and limit carbohydrates in the diet (OR = 1.39; 95% CI: 1.1–1.75). The group with a healthy weight was distinguished by eliminating products containing preservatives/artificial colors (OR = 0.76; 95% CI: 0.63–0.91) and performing home exercises, such as yoga or Pilates (OR = 0.68; 0.55–0.84). The obese group relative to the overweight group was more likely to perform regular exercise at least three times a week (OR = 1.5; 95% CI: 1.09–2.06), taking care for the presence of dietary fiber in the daily diet (OR = 1.35; 95% CI: 1.04–1.75) but also not paying much attention to one’s diet (OR = 1.23; 95% CI: 1.01–1.5). **Conclusions**: This study pointed out differences in lifestyle, especially food-related behaviors and physical activity among different BMI groups.

## 1. Introduction

Excess body weight is a global public health problem [1,2]. Overweight and obesity are characterized by excessive fat deposits (globally, regionally, and in organs as ectopic lipids) that increase the risk for adverse health outcomes [2]. The World Health Organization (WHO) estimates that, in 2022, approximately 43% of adults (≥18 years) were overweight and 16% were living with obesity [2]. Since 1980, the worldwide prevalence of overweight and obesity has doubled [3]. Body mass index (BMI) that is based on anthropometric measures ((kg)/height^2^ (m^2^)) is a surrogate marker of fatness [4]. In adults, BMI can be used to classify individuals into the following categories: underweight (BMI < 18.5), healthy weight (BMI 18.5–24.9), overweight (BMI 25–29.9), and obesity (BMI ≥ 30) [4,5]. BMI is often used in epidemiological and cross-sectional studies to identify individuals with excess body weight [4,5].

The imbalance of energy intake (diet) and energy expenditure (physical activity) is the major cause of overweight and obesity [2,6]. Lifestyle-related behaviors play a crucial role in the development of overweight and obesity [6,7,8]. A sedentary lifestyle and unhealthy diet are lifestyle patterns often observed in people with overweight or obesity [7,8]. However, genetic factors, psycho-social factors, and obesogenic environments also contribute to the risk of overweight and obesity development [7].

Excess energy intake resulting from nutrition-related behaviors is a key driver in gaining weight [9]. The type of food consumed, the caloric value of meals, the diversity of the diet, the balancing of the diet, the inclusion of vegetables and fruits, the elimination of selected groups of food products, avoiding highly processed foods, and limiting the consumption of sugars and fats are the basic food-related behaviors influencing the risk of overweight and obesity [10,11]. In addition, the number of meals, the regularity of their consumption, the conditions in which meals are consumed, and the time allocated for consumption are also behaviors that may influence the risk of excess energy intake [11,12].

Physical activity is a major form of energy expenditure that helps ensure energy balance [13,14]. Both everyday physical activities related to performing basic activities at home, at work, and while moving around as well as moderate or intense physical effort undertaken intentionally (“playing sports”) are forms of physical activity that can reduce the risk of being overweight or obese [14,15].

Excess body mass is associated with a higher risk of numerous diseases, including cardiovascular diseases, diabetes, cancers, respiratory diseases, neurological disorders, musculoskeletal diseases, and digestive disorders [16,17,18]. Obesity is associated with a lower quality of life and often with difficulties in social life and the risk of stigma or discrimination [17]. Excess body weight is associated with higher medical expenditures [18]. It is estimated that treating obesity-related issues accounts for 8.4% of total healthcare spending in OECD countries [19]. 

Numerous public health strategies promote healthy lifestyles and prevent overweight and obesity [20,21,22]. Regulation of the food market, reducing the fat, sugar, and salt content in food, ensuring access to healthy and nutritious food choices, restricting marketing of processed/high-sugar food, sin taxes, nutrition education as well as actions supporting regular physical activity are implemented worldwide [21,22,23,24]. However, the effectiveness of lifestyle-related interventions is varied [24].

A better understanding of lifestyle-related behaviors may help in planning and implementing more effective public policies and interventions [25]. Understanding differences in food-related behaviors and physical activity among groups with healthy weight, overweight, and obesity may inform healthcare professionals and policymakers on actions and goals that should be set to address the problem of excess body weight in the population [26].

Poland is a European country with 37.5 million citizens (fifth most populous country in the European Union), where over half of adults (56.6%) have excessive body weight (BMI > 25 kg/m^2^) [27]. Rapid economic development and socio-cultural changes observed in the past three decades caused significant changes in lifestyle-related behaviors and placed Poland among the European countries with a high burden of overweight and obesity as well as population aging [28]. The prevention of overweight and obesity is one of five operational goals of the National Health Program in Poland [29]. Epidemiological data from Poland can be helpful for other countries in Central and Eastern Europe that face similar socioeconomic changes and lifestyle behaviors, resulting from similar historical and social backgrounds.

This study aimed to identify differences in food-related behaviors and physical activity among healthy weight, overweight, and obese adult individuals in Poland. Particular attention was paid to health inequalities among the analyzed groups.

## 2. Materials and Methods

### 2.1. Data Source and Study Participants

Data for this secondary analysis were received from the dataset managed by the National Centre for Health Policy and Health Inequalities of the Cardinal Stefan Wyszynski University. Under a contract with the Polish Ministry of Education and Science (Agreement No. MEiN/2023/DPI/2717 of 13 October 2023), the Centre collected data on health-related behaviors and health inequalities in the Polish population [30] that were publicly communicated in the report “Health prevention and health inequalities” [30].

For the purposes of this study, data on seven questions used in a public opinion survey on attitudes and behaviors towards health prevention were driven from the database managed by the Centre. Originally gathered data came from the representative nationwide sample of >5000 adults (18–64 years) carried out in December 2024 using an IT research system (computer-assisted web interview) managed by a public opinion survey company (ARC Rynek i Opinia) [31]. Data were received free of charge, under the statutory activity of the Centre for Health Policy and Health Inequalities, as a part of public information activities and data sharing in publicly funded programs.

According to the data published by the Centre, the stratification model included: gender, age, size of the place of residence, as well as level of education based. Study sample was calculated based on the data published by the Statistics of Poland, and the demographic structure was adjusted to adult population of Poland of working age (18–64 years). Participation in the survey was voluntary. Voluntary and informed consent was expressed by all the participants. The study protocol was approved by the Ethical Committee at the Medical University of Warsaw (decision number: AKBE/56/2025).

### 2.2. Measures

Based on the data available in the dataset gathered and managed by the National Centre for Health Policy and Health Inequalities, query on seven records (questions) was submitted. The following questions (including multiple-answer questions) were included in this secondary analysis:

- How often do you check your weight (weigh yourself)?
- Which of the following methods do you use to track physical activity and diet?
- How many meals do you usually eat in a day?
- Which of the following statements best describes your daily eating habits?
- Which of the following statements best describes your physical activity?
- During the week (7 days), how much time do you usually spend in total on moderate physical activity such as cycling or walking?
- During the week (7 days), how much time do you usually spend in total on vigorous physical activity such as running?

**Figure 1:**
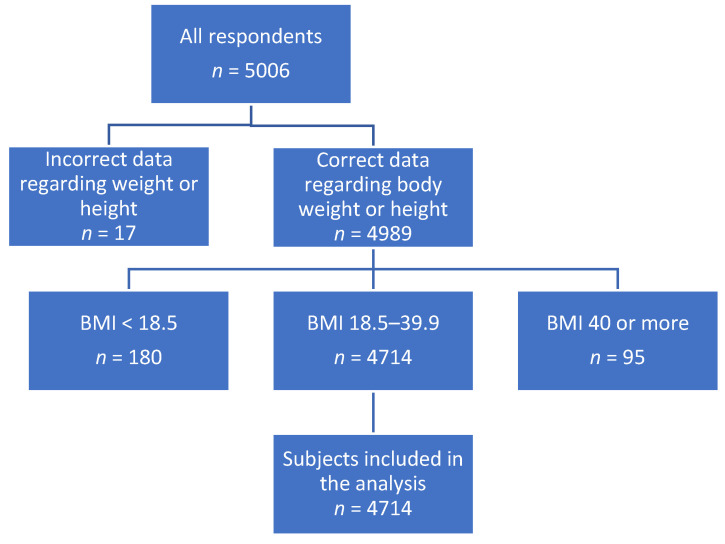
Respondents flow chart.

A total of 36 different lifestyle-related behaviors were analyzed. Moreover, data on demographic characteristics were obtained. Lifestyle-related behaviors were self-declared and there was no verification through direct visits to the subjects or weighing and analyzing the meals consumed in given periods. Based on the weight and height (self-performed measure in the last 7 days) that was used to calculate BMI, respondents were classified into 3 groups: healthy weight (BMI 18.5–24.9), overweight (BMI 25–29.9), and obesity (BMI 30–39.9). In total, records on 4714 adults were included in this secondary data analysis (Figure 1).

### 2.3. Data Analysis

The electronic database was prepared based on data driven from the dataset gathered and managed by the Centre for Health Policy and Health Inequalities [30]. Descriptive data were presented with frequencies and proportions. The chi-square test was used to analyze differences among qualitative variables. Multivariate logistic regression models predicting assignment to the group with healthy weight or overweight (Model 1) or overweight group vs. obesity group (Model 2) were prepared. The selection of variables for the models was based on the backward elimination method using the likelihood ratio. The model quality assessment was calculated based on the Cox and Snell R-square and the Nagelkerke R-square values. Statistical significance level was set at *p* < 0.05.

## 3. Results

### 3.1. Participants—Descriptive Characteristics

A total of 4714 adults were included into the analysis, of which 47.0% had healthy weight (BMI 18.5–24.9), 34.2% were overweight (BMI 25–29.9), and 18.9% were obese (BMI 30–39.9). The analyses excluded the underweight group (BMI < 18.5) and the morbidly obese group (BMI 40 and over). In the study group, men constituted 51.2%. The average age was 42.2 years (STD = 12.6) with a median of 42 years. In the group of men, the average age was 41.7 years (STD = 12.6), with a median of 41. Among women, the average age was 42.1 years (12.6) and the median was 43 years. The largest group were people with a secondary education (30.6%). Rural residents constituted 40.6% of the respondents. Detailed data are presented in Table 1.

### 3.2. Physicial Activity and Diet Monitoring Among Adults in Poland

Among all subjects (*n* = 4714), 12.9% checked their weight a few times a week. Another 34.6% checked it a few times a month, and 34.4% a few times a year. A total of 6.1% of the respondents declared weight measure once a year, and 7.4% declared weight measure less often than once a year. The group that did not measure their weight at all consisted of 4.6%. No statistically significant differences were observed depending on the BMI group (*p* = 0.062). Only after considering gender in the analysis did BMI begin to differentiate behaviors (in both groups *p* < 0.01).

**Figure 2:**
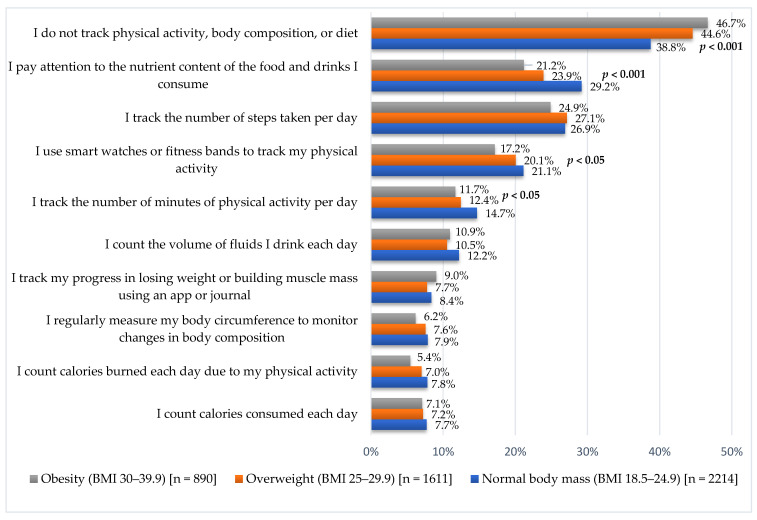
Actions taken to track physical activity and diet depending on BMI (multiple-choice question—respondents could indicate more than one answer).

Among all respondents, 42.2% declared that they did not monitor physical activity, body composition, and diet (Figure 2). The highest percentage of such declarations was in the group with obesity (46.7%), and the lowest was in the group with healthy weight (38.8%; *p* < 0.001). The three most frequently mentioned actions taken by respondents to monitor physical activity and diet were: tracking the number of steps taken during the day (26.6%); paying attention to the content of nutrients in consumed food and drinks (25.9%); and using smart watches or fitness bands to track physical activity (20.0%). In the group with healthy weight, a higher percentage of people (14.7%) monitoring the number of minutes of physical activity was observed compared to the overweight (12.4%) and obese (11.7%; *p* < 0.05). People with a healthy weight significantly more often pay attention to the content of nutrients in the food and drinks they consume (29.2% vs. 23.9% among those with overweight and 21.1% among those with obesity; *p* < 0.001), and similarly in the case of using smart watches or fitness bands to track physical activity (*p* < 0.05). In the group with healthy weight, there were 21.1% of such people, 20.0% overweight, and 17.2% obese (Figure 2). There were no statistically significant differences (Figure 2) by BMI category for: monitoring daily steps (*p* = 0.427); counting fluid intake (*p* = 0.251); tracking progress in losing weight or building muscle mass (*p* = 0.549); regularly measuring body circumference (*p* = 0.264); counting calories consumed each day (*p* = 0.758); or counting calories resulting from physical activity (*p* = 0.060).

### 3.3. Diet-Related Habits Among Adults in Poland

**Figure 3:**
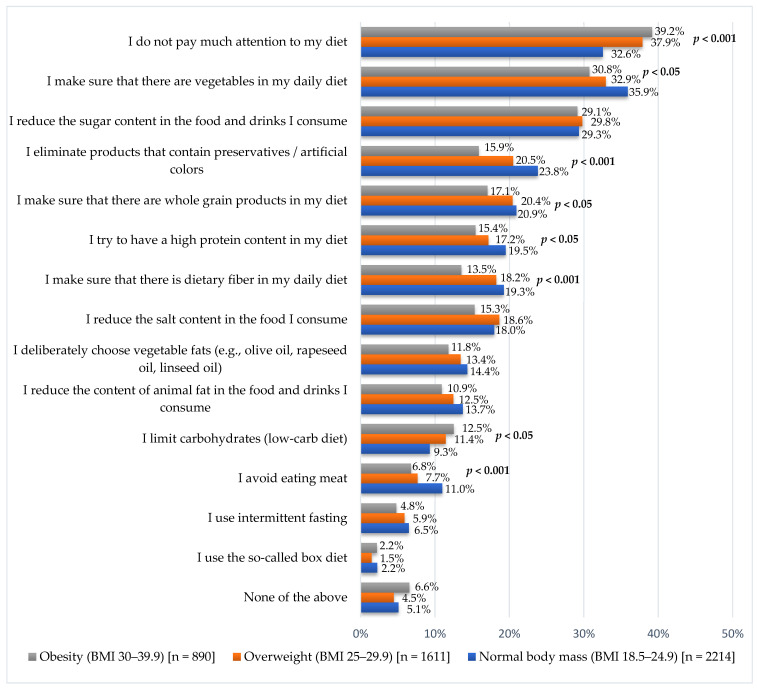
Diet-related behaviors depending on BMI (multiple-choice question—respondents could indicate more than one answer).

Among the subjects (*n* = 4714), 10.1% declared eating five or more meals a day. Four meals a day were consumed by 32.9%, and three meals a day were consumed by 43.8% (Figure 3). According to the declarations, two meals a day were consumed by 11.4% of the subjects, and one meal a day was consumed by 1.7%. There were no statistically significant differences by BMI groups (*p* = 0.408). Also, when divided by gender, the BMI group did not differentiate the results (in the group of men, *p* = 0.122; in the group of women, *p* = 0.370).

In this study (Figure 3), 35.6% of subjects declared that they did not pay attention to their diet. The BMI group statistically significantly differentiated the results (*p* < 0.001). In the group with healthy weight, this percentage was 32.6%; in the group with overweight, this percentage was 37.9%; and in the group with obesity, this percentage was 39.1%. The three most frequently mentioned diet-related behaviors were: ensuring the presence of vegetables in the daily diet (33.9%); reducing the sugar content in consumed food and drinks (29.5%); and eliminating products that contain preservatives/artificial colors (21.2%).

Consumption of an adequate proportion of vegetables (Figure 3) was declared by 35.9% of people with healthy weight, 33.0% with overweight, and 30.8% with obesity (*p* < 0.05). In the group with healthy weight, 23.8% ensured the elimination of products that contain preservatives or artificial colors. Among those with overweight, this was 20.5%, and among those with obesity, it was 15.9% (*p* < 0.001). In the group with healthy weight, 20.9% indicated that they ensured the presence of whole grain products in the diet. In the overweight group, it was 20.4%, and in the obese group, it was 17.1% (*p* < 0.05).

In this study, 19.5% of people with healthy weight declared attempts to have a high protein content in their diet. Among overweight subjects, it was 17.2%, and in the obese group, it was 15.4% (*p* < 0.05). The presence of dietary fiber in the daily diet was taken care of by 19.2% of people with healthy weight, 18.2% of overweight, and 13.5% of obese (*p* < 0.001). Limiting the consumption of carbohydrates was declared by 9.3% of people with healthy weight. Among those overweight subjects, it was 11.4%, and in the obese group, it was 12.5% (*p* < 0.05). In the group with healthy weight, 11.0% avoided eating meat. Among overweight subjects, it was 7.7%, and in the obese group, it was 6.7% (*p* < 0.001).

No statistically significant differences were observed concerning BMI categories in the cases of: reduction in sugar content in consumed food and beverages (*p* = 0.927); reduction in salt content (*p* = 0.101); deliberate choice of vegetable fat (*p* = 0.164); reduction in animal fat content in consumed food and beverages (*p* = 0.095); use of intermittent fasting 6.0% (*p* = 0.203); and use of the so-called box diet (*p* = 0.204). Detailed data are presented in Figure 3.

### 3.4. Physicial Activity Undertaken by Adults in Poland

Among all subjects (*n* = 4714), 16.5% reported no moderate or vigorous physical activity during the week. Results varied by BMI group (*p* < 0.001). Among those with healthy weight, 13.6% reported no such physical activity, while among overweight individuals, this was 17.0%, and obese individuals, 22.8%. Gender differences increased the differences in results in the women group. Among healthy weight women, no moderate or vigorous physical activity was reported by 14.2%, while in the obese group, this was 27.0% (*p* < 0.001). In the men’s group, this was 13.0% vs. 19.8%, respectively (*p* < 0.01).

When asked about specific physical activities, 20.8% of healthy weight, 26.0% of overweight, and 35.9% of obese respondents indicated that they did not perform any form of physical activity (*p* < 0.001).

The three most frequently mentioned forms of physical activity were: regular walking or cycling (34.5%); spending time outdoors (running or hiking) (23.8%); and exercising at home (yoga or Pilates) (13.7%). Regular walking or cycling was reported by 36.7% of healthy weight, 35.6% of overweight respondents, and 27.3% among obese subjects (*p* < 0.001). The preferred outdoor activity (running or hiking) was indicated by 25.5% of healthy weight, 23.9% of overweight, and 19.1% of obese respondents.

**Figure 4:**
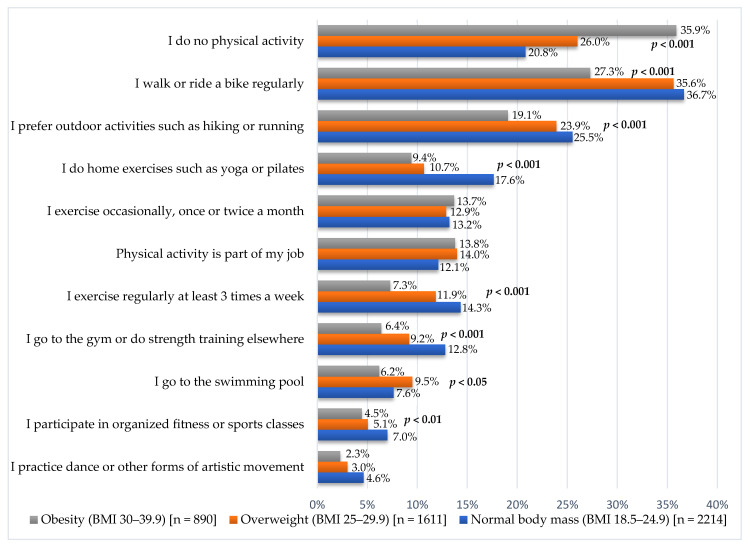
Physical activity undertaken by adults in Poland depending on BMI (multiple-choice question—respondents could indicate more than one answer).

Home exercises such as yoga or Pilates were performed by 17.7% of the healthy weight group. In the overweight group, it was 10.7%, and in the obese group, 9.4% (*p* < 0.001). Sports were practiced at least three times a week by 14.4% of the healthy weight group, 11.9% of the overweight group, and 7.3% of the obese group (*p* < 0.001). Among the healthy weight group, 12.8% declared going to the gym or doing strength training. In the overweight group, it was 9.2%, and in the obese group, 6.4% (*p* < 0.001). A swimming pool was used by 7.6% of the healthy weight group, 9.5% of the overweight group, and 6.2% of the obese group (*p* < 0.05). Among the healthy weight group, 7.0% declared participation in organized fitness or sports classes. In the overweight group, this percentage was 5.0%, and in the obese group, 4.5% (*p* < 0.01). Dance or other forms of artistic movement were indicated by 4.7% of people with healthy body weight, 3.0% of overweight people, and 2.2% of obese people (*p* < 0.01). No statistically significant differences were observed concerning the BMI category in the case of occasional exercise (*p* = 0.850) and people who declared that physical activity was part of their professional work (*p* = 0.185). Details are presented in Figure 4.

### 3.5. Factors Associated with Nutrition-Related Behaviors and Physical Activity

The multivariate logistic regression model predicting assignment to the group with healthy weight (BMI 18.5–24.9) or overweight (BMI 25–29.9) obtained the Cox and Snell R-square of 0.104 and the Nagelkerke R-square of 0.140 (*n* = 3825). Among the variables included in the model, the factor most strongly associated with overweight (vs. healthy weight) was age. People aged 45–64 had almost 6 times higher odds of belonging to the overweight group compared to people aged 18–24 (OR = 5.7; 95% CI: 4.34–7.49). In the case of the 35–44 age group, the odds were 3.5 times higher (OR = 3.55; 95% CI: 2.69–4.7), and for the 25–34 age group, the odds were almost twice as high (OR = 1.94; 95% CI: 1.45–2.6). Men were more likely to be overweight (OR = 1.69; 95% CI: 1.47–1.95), as were rural residents (OR = 1.21; 95% CI: 1.05–1.39). Among the lifestyle factors and health-promoting behaviors, the overweight group was more likely to monitor the number of steps taken during the day (OR = 1.42; 95% CI: 1.19–1.71) and limit carbohydrates in the diet (OR = 1.39; 95% CI: 1.1–1.75). In turn, the group with healthy weight was distinguished by eliminating products containing preservatives/artificial colors (OR = 0.76; 95% CI: 0.63–0.91) and performing home exercises, such as yoga or Pilates (OR = 0.68; 0.55–0.84). Detailed data are presented in Table 2.

The multivariate logistic regression model predicting assignment to the overweight (BMI 25–29.9) or obese (BMI: 30–39.9) group obtained the Cox–Snell R-square of 0.029 and the Nagelkerke R-square of 0.040 (*n* = 2501). Among the variables included in the model, the behaviors characterizing the obese group (relative to the overweight group) were in particular regular exercise at least three times a week (OR = 1.5; 95% CI: 1.09–2.06) and taking care of the presence of dietary fiber in the daily diet (OR = 1.35; 95% CI: 1.04–1.75) but also not paying much attention to one’s diet (OR = 1.23; 95% CI: 1.01–1.5). In turn, the overweight group (vs. obese) was distinguished by the lack of physical activity (0.62; 95% CI 0.5–0.75) or tracking progress in losing weight or building muscle mass using an application or a diary (OR = 0.67; 95% CI: 0.49–0.92). Detailed data are presented in Table 3.

## 4. Discussion

This is one of the most up-to-date and comprehensive studies on food-related behaviors and physical activity that was carried out in a representative sample of adults of working age (18–64 years). Findings from this study showed that people with healthy weight, compared to those with overweight or obesity, more often track physical activity, body composition, or diet; pay more attention to the nutrient content of food and drinks; eliminate selected groups of meals; and pay more attention to the intake of vegetables, protein, fiber, and whole grains. Moreover, people with healthy weight, when compared to overweight or obese individuals, more often perform physical activity like walking, riding a bike, running, and yoga or Pilates or go to a gym or fitness or organized sports classes. In multivariable logistic regression, age was the most important factor associated with being assigned to an overweight group when compared to a healthy weight group (*p* < 0.05). Individuals with obesity compared to those with overweight were more likely to not pay attention to diet, including dietary fiber in diet, and exercise regularly at least 3 times a week (*p* < 0.05).

In this study, habits related to monitoring diet and physical activity were assessed. Findings from this study showed that people with healthy weight (BMI 18.5–24.9) more often declared tracked physical activity, body composition, or diet when compared to those with excess body mass. Frequent dietary tracking is important for consistent long-term weight loss [32]. In recent years, wearables and mobile apps have gained popularity as tools to monitor physical activity and diet [33]. However, in this study, people with excess body weight less often declared the use of those new technologies when compared to people with healthy weight. This observation points out that the potential of smart devices and mobile apps is not fully used when related to diet and physical activity monitoring.

Several food groups like refined grains, red meat, and sugar-sweetened beverages are associated with an increased risk of adiposity [34], whereas the intake of whole grains, vegetables, and fruits is linked with reduced weight gain [34]. Findings from this study showed that people with excess body weight compared to those with healthy weight are less likely to track physical activity, body composition, or diet. Moreover, people with healthy weight more often declared the consumption of vegetables, dietary fiber, whole grains, and high protein and avoided eating meat as well as eliminating products that contain preservatives/artificial colors. However, people with excess body weight compared to those with healthy weight more often declared limiting carbohydrates (low-carb diet). These data contribute to the current state of knowledge on food-related habits of people assigned to different groups based on BMI. Individuals with healthy weight more often consumed groups of food products that are linked with a lower risk of weight gain like whole grains and vegetables [35,36]. Moreover, our observation on the limited consumption of products that contain preservatives or artificial colors may point out the dietary trends resulting from nutrition education but also food marketing [37,38]. We can hypothesize that people with healthy weight are more likely to choose ecological or bio food products and this consumer behavior may result from the pro-healthy marketing of these products.

Physical activity is necessary to keep energy balance and is a key form of energy expenditure [39]. The effectiveness of different forms of physical activity in weight loss is varied [14,40]. However, regardless of the type of activity, regular physical activity is essential for weight maintenance [40]. In this study, going to a swimming pool was the only type of physical activity that was more popular among people with excess body weight when compared to the healthy weight group. Swimming and aquatic exercise, due to the physiology of sports and the impact of water buoyancy on lower joint load than, for example, running, may be considered as better-tolerated forms of physical activity among those with excess body weight [41]. This observation points out that local investments in sports infrastructure like swimming pools may be considered as a part of strategies aimed at weight control and management at the population level. Findings from this study showed that people with healthy weight when compared to overweight or obese individuals more often perform physical activity like walking, riding a bike, running, or yoga or Pilates or go to a gym or fitness or organized sports classes. This observation may result from the fact that the abovementioned types of physical activity may be related to greater body load, effort, and fatigue in overweight and obese people than in people with healthy weight due to the pressure of one’s body weight on the limbs and the individual experiences associated with it [15].

This study also focused on potential health inequalities related to three different groups of people defined based on BMI. In this study, 13 different factors significantly associated with assignment to the overweight group than to the healthy weight group were identified. Age was the most important factor associated with being overweight. This observation is in line with previous reports on weight change across adulthood [42]. Men were more likely to be overweight, which is also in line with global epidemiological data on the gender differences in the prevalence of overweight and obesity [1,2]. In this study, living in rural areas and having less than a higher education were associated with a higher risk of being overweight. This observation points out potential socioeconomic differences that contribute to the risk of being overweight and should be considered in analyses of health inequalities. When compared to those with healthy weight, people with overweight were more likely to limit carbohydrates and reduce the sugar content in their food or drink. This observation suggests that people with overweight implemented basic food modification to reduce or control weight, mostly related to sugar consumption [43]. Further nutritional education activities are needed to improve nutritional knowledge among those with excess body weight. There was no significant impact of gender on lifestyle-related behaviors in these groups, which suggests that there are no extensive gender differences in lifestyle-related behaviors that lead to overweight.

In this study, seven different factors significantly associated with assignment to the obesity group than to the overweight group were identified. People with obesity were more likely to exercise regularly at least three times a week and pay attention to consuming dietary fiber. Fiber in diet is known as a food that may help in weight loss and reduce obesity-related disorders [44]. However, people with obesity compared to those with overweight were less likely to track progress in losing weight using an app or journal and were more likely to not pay attention to diet. This observation suggests the need to educate people with obesity on the role of excess body weight as a risk factor for numerous diseases and adverse health effects. There were no impact gender differences in lifestyle-related behaviors between overweight and obese individuals, which suggests lifestyle-related education and key messages targeted to people with excess body weight should not be differentiated by gender.

This study has several practical implications for healthcare professionals and policymakers. The data presented in this study provided scientific evidence on differences in lifestyle-related habits (especially nutrition and physical activity) among individuals with healthy weight, overweight, and obesity. Differences in nutrition-related behaviors presented in this study may be used by healthcare professionals to plan and develop further educational campaigns, especially those targeted to individuals with excess body weight. Moreover, data on physical activity undertaken by different groups based on BMI values may be used to promote types of physical activity among people with excess body weight as well as by policymakers to provide access to infrastructure adjusted to the preferences of people with overweight or obesity. Moreover, this study underlines the role of age and educational level in shaping lifestyle-related behaviors related to diet and physical activity and points out the need to target people without higher education and older adults in public health interventions.

This secondary data analysis has several limitations. The scope of the analysis was limited to the data acquired by the institution managing the dataset. The dataset acquired for this study was limited to seven questions derived from the nationwide cross-sectional survey carried out with the CAWI technique in December 2024. The study population included only adults of working age (18–64 years), so this study is not representative of the complete adult population of Poland. Data on weight and height were self-reported, but respondents were instructed on how to perform measures. Self-reported data were used to calculate BMI, and medical records and additional measures by healthcare professionals were not collected. Data on lifestyle-related habits like nutrition and physical activity were also self-declared, so some form of recall bias may occur. There were no questions on the amount (weight) of particular nutrients, and questions on habits and behaviors (like regular consumption or consumption of adequate amount of nutrients) were self-reported based on the individual opinion of respondents. In this study, the underweight and morbidly obese groups were not included. The benefit of including them would be to obtain a picture of the entire population. However, in in-depth analyses, due to their small number, it would be necessary to combine them with other categories, which would disturb the clarity of inference.

## 5. Conclusions

This study pointed out differences in lifestyle, especially food-related behaviors and physical activity among different BMI groups: individuals with healthy weight, overweight, and obesity. Differences in the characteristics of food products consumed by different BMI groups pointed out the need for further directions of nutrition education in Poland. Moreover, factors associated with assignment to overweight or obesity groups that were presented in this study suggest that there are health inequalities and gaps in health literacy levels by body mass index, and further actions are needed to better address these inequalities. Data on lifestyle, especially food-related habits and physical activity among adults of working age, should be included in further educational campaigns and policy papers to better adjust local policies and interventions to gaps in knowledge on healthy lifestyles among people with excess body weight.

## Figures and Tables

**Table 1:** Description of the study population.

	Healthy Weight (BMI 18.5–24.9)	Overweight (BMI 25–29.9)	Obesity (BMI 30–39.9)	Overall
	*n* = 2214	*n* = 1611	*n* = 890	*n* = 4714
Gender				
men	992 (44.8)	915 (56.8)	505 (56.8)	2412 (51.2)
women	1221 (55.2)	696 (43.2)	384 (43.2)	2301 (48.8)
Age				
18–24	355 (16)	82 (5.1)	42 (4.7)	479 (10.2)
25–34	564 (25.5)	238 (14.8)	113 (12.7)	915 (19.4)
35–44	586 (26.5)	432 (26.8)	241 (27)	1259 (26.7)
45–64	709 (32)	860 (53.3)	495 (55.6)	2064 (43.8)
Educational level				
primary or vocationals	598 (27)	543 (33.7)	340 (38.2)	1481 (31.4)
secondary	829 (37.4)	608 (37.7)	352 (39.6)	1789 (38.0)
higher	787 (35.5)	460 (28.6)	197 (22.2)	1444 (30.6)
Place of residence				
Rural area	838 (37.9)	674 (41.8)	400 (45)	1912 (40.6)
city <100,000 residents	724 (32.7)	507 (31.5)	273 (30.7)	1504 (31.9)
city 100,000–499,999 residents	365 (16.5)	251 (15.6)	121 (13.6)	737 (15.6)
city ≥ 500,000 residents	287 (13)	179 (11.1)	95 (10.7)	561 (11.9)

**Table 2:** Multivariate logistic regression model predicting assignment to the healthy weight group (BMI 18.5–24.9) vs. overweight group (BMI 25–29.9); *n* = 3825.

	Factors Associated with Assigment to Overveight Group		
Variable	Response	*p*	OR (95% CI)
I do not track physical activity, body composition, or diet	yes	*p* < 0.05	1.22 (1.02–1.45)
	no	Ref.	Ref.
I track the number of steps I take during the day	yes	*p* < 0.001	1.42 (1.19–1.71)
	no	Ref.	Ref.
I track my progress in losing weight or building muscle mass using an app or journal	yes	*p* < 0.05	1.3 (1–1.7)
	no	Ref.	Ref.
I reduce the sugar content in the food and drinks I consume	yes	*p* < 0.05	1.21 (1.02–1.44)
	no	Ref.	Ref.
I avoid eating meat	yes	*p* = 0.059	0.79 (0.62–1.01)
	no	Ref.	Ref.
I limit carbohydrates (low-carb diet)	yes	*p* < 0.01	1.39 (1.1–1.75)
	no	Ref.	Ref.
I eliminate products that contain preservatives/artificial colors	yes	*p* < 0.01	0.76 (0.63–0.91)
	no	Ref.	Ref.
I do no physical activity	yes	*p* < 0.05	1.21 (1.02–1.44)
	no	Ref.	Ref.
I do home exercises such as yoga or pilates	yes	*p* < 0.001	0.68 (0.55–0.84)
	no	Ref.	Ref.
I go to the swimming pool	yes	*p* < 0.05	1.33 (1.04–1.71)
	no	Ref.	Ref.
Gender	men	*p* < 0.001	1.69 (1.47–1.95)
	women	Ref.	Ref.
Education	vocational	*p* < 0.05	1.27 (1.05–1.53)
	secondary	*p* < 0.01	1.24 (1.06–1.45)
	other	Ref.	Ref.
Age	18–24 years	Ref.	Ref.
	25–34 years	*p* < 0.001	1.94 (1.45–2.6)
	35–44 years	*p* < 0.001	3.55 (2.69–4.7)
	45–64 years	*p* < 0.001	5.7 (4.34–7.49)
Location of the place of residence	rural	*p* < 0.01	1.21 (1.05–1.39)
	urban	Ref.	Ref.

**Table 3:** Multivariate logistic regression model predicting assignment to the overweight group (BMI 25–29.9) vs. obesity group (BMI 30–39.9); *n* = 2501.

	Factors Associated with Assigment to Obesity Group		
Variable	Response	*p*	OR (95% CI)
I track my progress in losing weight or building muscle mass using an app or journal	yes	*p* < 0.05	0.67 (0.49–0.92)
	no	Ref.	Ref.
I don’t pay much attention to my diet	yes	*p* < 0.05	1.23 (1–1.5)
	no	Ref.	Ref.
I limit carbohydrates (low-carb diet)	yes	*p* < 0.05	0.74 (0.56–0.97)
	no	Ref.	Ref.
I make sure that my daily diet includes dietary fiber	yes	*p* < 0.05	1.35 (1.04–1.75)
	no	Ref.	Ref.
I eliminate products that contain preservatives/artificial colors	yes	*p* = 0.054	1.27 (1–1.62)
	no	Ref.	Ref.
I don’t do any physical activity	yes	*p* < 0.001	0.62 (0.5–0.75)
	no	Ref.	Ref.
I exercise regularly at least 3 times a week	yes	*p* < 0.05	1.5 (1.09–2.06)
	no	Ref.	Ref.
I exercise occasionally, once or twice a month	yes	*p* = 0.055	0.78 (0.6–1.01)
	no	Ref.	Ref.
Education	primary	*p* < 0.001	0.51 (0.35–0.72)
	vocational	*p* < 0.05	0.78 (0.62–0.99)
	secondary	*p* < 0.01	0.74 (0.6–0.92)
	higher	Ref.	Ref.

## Data Availability

This study is a secondary data analysis. Dataset is available upon request to the authors due to legal reasons.
